# Impact of *CCND1* amplification on the prognosis of hormone receptor–positive, HER2-negative breast cancer patients—correlation of clinical and pathological markers

**DOI:** 10.1007/s10549-024-07545-x

**Published:** 2024-11-26

**Authors:** Dorothea Hanf, Peter Fasching, Paul Gass, Matthias W. Beckmann, Carolin C. Hack, Felix Heindl, Lothar Häberle, Nelson John, Ramona Erber, Michael F. Press, Matthias Rübner, Patrik Pöschke

**Affiliations:** 1 Department of Translational Medical Oncology, National Center for Tumor Diseases Dresden (NCT/UCC) and Helmholtz-Zentrum Dresden-Rossendorf (HZDR), Dresden, Germany; 2 Department of Gynecology and Obstetrics, Erlangen University Hospital, Comprehensive Cancer Center Erlangen-EMN (CCC ER-EMN) / Friedrich Alexander University of Erlangen–Nuremberg (FAU), Universitätsstrasse 21–23, 91054 Erlangen, Germany; 3 Biostatistics Unit, Erlangen University Hospital, Department of Gynecology and Obstetrics, Comprehensive Cancer Center Erlangen-EMN, Friedrich-Alexander-Universität Erlangen-Nürnberg, Erlangen, Germany; 4 Translational Medical Oncology, Faculty of Medicine, Carl Gustav Carus University Hospital, TUD Dresden University of Technology, Dresden, Germany; 5 Partner Site Dresden, German Cancer Consortium (DKTK), Heidelberg, Germany; 6 Institute of Pathology, Erlangen University Hospital, Comprehensive Cancer Center Erlangen-EMN (CCC ER-EMN) / Friedrich Alexander University of Erlangen–Nuremberg (FAU), Erlangen, Germany; 7 Department of Pathology, Norris Comprehensive Cancer Center, University of Southern California, Los Angeles, CA USA; 8 Comprehensive Cancer Center Alliance WERA (CCC), Erlangen, Germany; 9 Bavarian Center for Cancer Research (BZKF), Erlangen, Germany

**Keywords:** Amplification, Breast cancer, *CCND1*, FISH, Prognosis

## Abstract

**Purpose:**

The cyclin D1 gene (*CCND1*) encodes a key cell-cycle regulatory protein. Resistance to endocrine therapy is reportedly observed more often in patients with *CCND1*-amplified tumors. *CCND1* amplification is known to be a driving event in breast cancer, but contradictory findings are reported for its association with prognosis. This study therefore investigated the prognostic value of *CCND1* amplification in hormone receptor (HR)-positive breast cancer patients.

**Methods:**

A cohort of 894 unselected breast cancer patients from the Bavarian Breast Cancer Cases and Controls (BBCC) study was included. The *CCND1* amplification rate was evaluated in tissue microarrays using fluorescence in situ hybridization. A *CCND1*/CEP11 ratio ≥ 2.0 was considered amplified. Statistical analysis was conducted on cases with ratios based on a range of 20–100 nuclei analyzed per case. A univariable Cox regression model was fitted with disease-free survival (DFS) and overall survival (OS).

**Results:**

*CCND1* gene status was assessable in 511 patients. The *CCND1* amplification rate was 12.9% (66 patients). Most patients with *CCND1* amplification had luminal B-Like—(51.5%, *n* = 34) or luminal A-Like tumors (25.8%, *n* = 17), 13 patients with HER2-positive disease (19.7%) and only two patients had triple-negative tumors (3.0%). Survival analysis, focused on HR-positive, HER2-negative patients, showed no statistically significant differences in the DFS and OS with and without *CCND1* amplification (*P* = 0.20 and 0.14, respectively, in the unadjusted analysis).

**Conclusions:**

*CCND1* amplification is a recurring event in breast cancer, occurring most frequently in luminal B-like and HER2-amplified subtypes. A trend toward less favorable outcomes was observed among *CCND1*-amplified HR-positive, HER2-negative tumors.

## Introduction

Breast cancer is the most common cancer among women, with three out of 10 cases affecting women younger than 55 years. It is therefore the entity that causes the greatest loss of lifetime among cancers [1].

Approximately 75% of breast cancers are estrogen receptor (ER)–positive. Hormone receptor (HR) positivity is the strongest predictor for hormonal therapy, which is a mainstay of breast cancer therapy [2]. Resistance to endocrine therapy is often equivalent to loss of tumor control, and despite new therapeutic strategies such as immunotherapy and cyclin-dependent kinase 4 and 6 (CDK4/6) inhibitors, breast cancer is still the most common cancer-related cause of death in women [3, 4].

Since HR-positive breast cancer is an entity with a highly variable somatic mutation landscape and diverse disease outcomes, biomarkers are needed in order to classify patients into a phenotypically benign group and contrasting subtypes that are prone to late recurrence, in order to enable risk-adapted therapy and more differentiated prognostic assessment.

Curtis et al. identified amplifications of the gene encoding cyclin D1 (*CCND1*), located on the long arm of chromosome 11.q13.3, as one of the most common driving mutations in breast cancer. It is one of the most commonly amplified and overexpressed genes at the protein level in breast cancer. *CCND1* amplifications mark a molecularly defined group of poor prognoses consisting of luminal carcinomas [5]. Cyclin D1 acts as a mitogen sensor, which allows the cell to pass the G1/S-phase restriction point [6]. Cyclin D1 oligomerizes with CDK 4/6, which enables E2F transcription factor activation, leading to the transcription of S-phase–relevant genes [7]. In the process, cyclin D1 is crucial for the progression of cells throughout the G1 phase of the cell cycle, which consequently becomes independent from further proliferative stimuli [8]. In epithelial cells of the breast, the expression of cyclin D1 is induced by estrogen. Cyclin D1 acts simultaneously as a ligand-independent activator of the estrogen receptor [9]. The central role of cyclin D1 in the cell cycle suggests oncogenic relevance, with higher cyclin D1 concentrations in the cell potentially leading to uncontrolled G1/S-point passage, facilitating the accumulation of further somatic mutations [10, 11]. As an estrogen-independent ER agonist, cyclin D1 may overcome the ER-blocking effects of antihormonal therapies.

Mouse experiments have demonstrated that there is a correlation between cyclin D1 overexpression and breast cancer formation [12]. However, noncanonical functions of cyclin D1 independent of CDK4/6 may contribute to oncogenesis [13].

Cyclin D1 amplification and overexpression are repeatedly found in HR-positive breast cancer and are assumed to play a central role in the development of endocrine therapy resistance [14, 15]. *CCND1* amplifications correlate with HR-positivity and luminal subtypes with amplification rates observed between 29 and 58% [16].

In view of these findings, *CCND1* status appears to have potential as a prognostic and predictive marker particularly in patents with HR-positive, human epidermal growth factor receptor 2 (HER2)-negative breast cancer, and it could be easily assessed in the clinical context. The purpose of this study was to determine the prognostic value of *CCND1* amplification and its correlation with clinically established biomarkers in patients with HR-positive HER2-negative primary breast cancer.

## Materials and methods

### Patient cohort

**Fig. 1:**
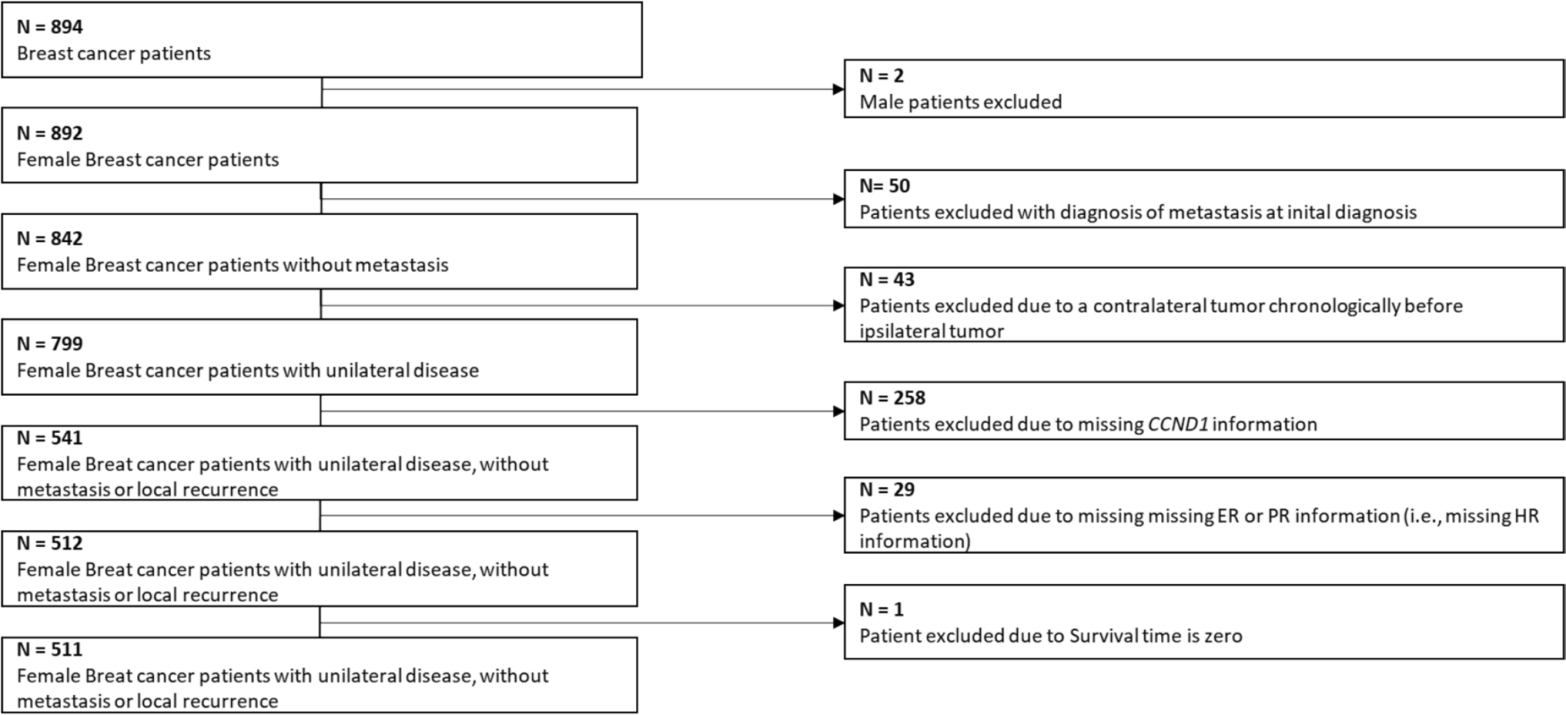
Patient selection

Breast cancer tissue was examined on tissue microarrays from 894 patients with primary invasive breast cancer, collected between 2002 and 2006 as part of the Bavarian Breast Cancer Cases and Controls (BBCC) Study. The BBCC study was a case–control study that initially included 1538 women with breast cancer, described in detail elsewhere [17], who received various treatments in accordance with University Breast Center guidelines at the University Breast Center for Franconia, which is part of Erlangen University Hospital (Bavaria, Germany). Tumor samples were collected from 1997 to 2007 [18, 19]. Approval for the study was obtained from the local ethics committee at the University of Erlangen (reference numbers 2700 and 297_17 Bc). The study was conducted in accordance with the “REMARK” recommendations [20]. Exclusion criteria used to define the final sample size in the present study of 511 patients were male sex, distant metastasis at initial diagnosis, survival time less than 1 day, local recurrence, or missing values for *CCND1* (Fig. 1).

### Collection of clinical and histopathological data

Data collection has been described in detail elsewhere [17]. Summarized, clinical, histopathological, and follow-up data were documented prospectively and annually as part of routine procedure in the University Breast Center for Franconia, as a certified breast center. The detailed methods used to assess these parameters have been described elsewhere [17]. Data on tumor type, grading, estrogen receptor (ER) status, progesterone receptor (PR) status, and HER2 status were obtained from the pathology files. Detailed information regarding the predictor molecular class can be found elsewhere [21].

### *Fluorescence *in situ* hybridization of CCND1*

**Fig. 2:**
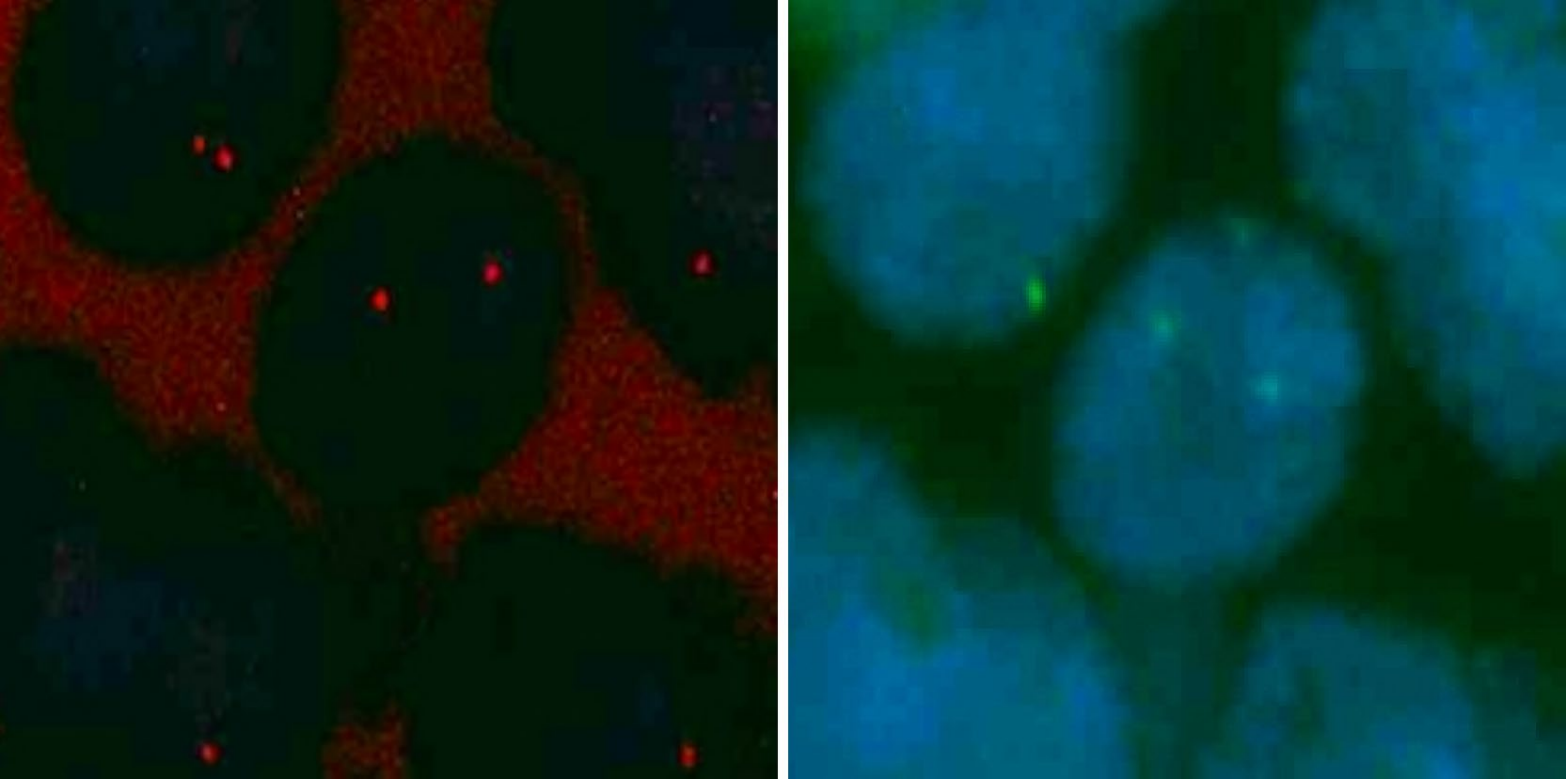
*CCND1* with fluorescence in situ hybridization (FISH). Tumor nuclei are marked using SpectrumOrange. The *CCND1* gene is depicted as an orange signal (left), and the centromere (CEP11) signal is green in a CCND1-negative cell (right)

Tissue microarrays (TMAs) of formalin-fixed, paraffin-embedded tumor tissue were built as described first by Kononen and coworkers in 1998 [22]. Fluorescence in situ hybridization (FISH) was carried out in accordance with the manufacturer’s recommendations and in-house standards. Fluorescence in situ analysis was performed using the Vysis® LSI (Locus Specific Identifier)® *CCND1* SpectrumOrange/CEP11 SpectrumGreen probes kit (all from Abbott Molecular, Des Plaines, Illinois, USA). The chromosome 11 centromere was determined using a SpectrumGreen-labeled chromosome enumeration probe (CEP11). The assay in the protocol used was tested and described in detail by Press and coworkers in 2002 [23]. The centromere gene probe CEP11 appeared as a green fluorescent signal, and the *CCND1*-LSI gene probe appeared as an orange fluorescent signal (Fig. 2).

**Fig. 3:**
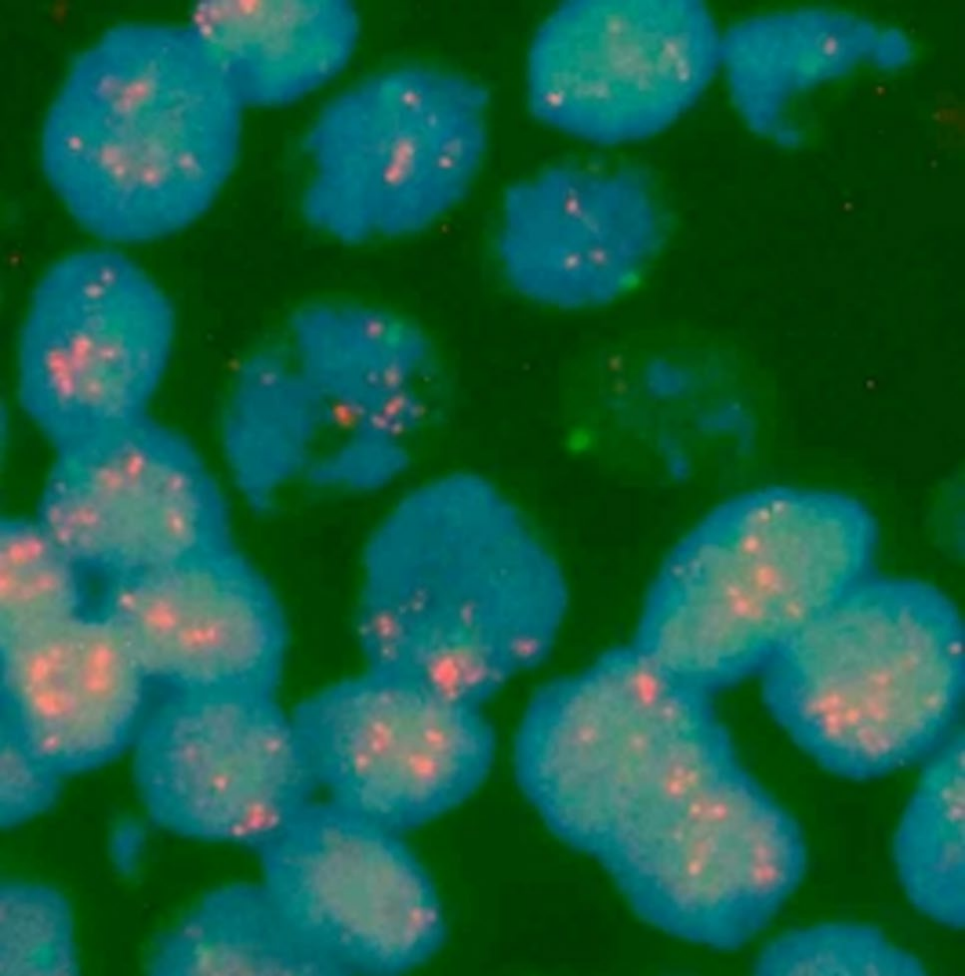
*CCND1* with fluorescence in situ hybridization (FISH). A *CCND1*-amplified probe with a *CCND1*/CEP11 ratio of 7.8

*CCND1* copy numbers were determined in a minimum of 20 interphase, nonoverlapping tumor cell nuclei and compared with CEP11 in the same nucleus, as recommended by the American Society of Clinical Oncology/College of American Pathologists (ASCO/CAP) guidelines used for FISH diagnosis for HER2-positive breast cancer [24, 25]. *CCND1* amplification was defined as a *CCND1* probe to CEP11 ratio of 2.0 or greater (Fig. 3). There are no validated suggested cutoff values for examining *CCND1* amplification using FISH analysis. The same ratio recommended by the U.S. Food and Drug Administration for HER2 amplification diagnosis for breast cancer was therefore used [26].

### Statistical analysis

Patient and tumor characteristics were described using appropriate summary statistics. Means with standard deviation are used for continuous characteristics, and frequency and percentage values for categorical characteristics.

Disease-free survival (DFS) was defined as the time from the date of primary diagnosis to the earliest date of disease progression (distant metastasis, local recurrence, contralateral breast cancer, death from any cause) or the date of censoring. Patients who were lost to follow-up before the maximum observation time of 10 years, or were disease-free after the maximum observation time, were censored at the last date on which they were known to be disease-free or at the maximum observation time. Overall survival (OS) was defined in a similar fashion.

The primary objective was to investigate whether *CCND1* status has prognostic value in HR-positive patients, in addition to well-known prognostic patient and tumor characteristics. A multivariable Cox regression model (the “basic model”) was fitted with DFS and OS as outcomes and the following predictors: Age at diagnosis (continuous), body mass index (continuous), tumor stage (ordinal, T1 to T4), lymph-node status (categorical; N0, N +) and grading (ordinal; G1, G2, G3). The proportional hazards assumptions were checked for both outcomes using the Grambsch and Therneau method [27]. Where the proportional hazards assumption was violated, stratification for the corresponding predictors was implemented in the models. On the basis of the results, the basic model for DFS was stratified by lymph-node status.

Subsequently, an additional Cox regression model (the “biomarker model”) was fitted, containing *CCND1* status (categorical; amplification yes/no) and the predictors from the basic model. The biomarker model was compared to the basic model using a likelihood ratio test (LRT). A significant *P* value would indicate that biomarker information improves the survival prognosis in addition to the other prognostic factors taken into account. An adjusted hazard ratio for *CCND1* was estimated using the biomarker model. An adjusted hazard ratio for *CCND1* amplification status with associated 95% confidence intervals (CI) and the *P* value from the Wald test were calculated using the biomarker model. Patients with missing survival data or a missing *CCND1* status were excluded from the analysis. Missing values in other predictors were imputed as in Salmen et al. [28].

For sensitivity analyses, an unadjusted hazard ratio was estimated using a univariable Cox regression model for *CCND1* status alone. Survival rates were estimated using the Kaplan‒Meier product limit method.

All of the tests were two-sided, and *P* < 0.05 was regarded as statistically significant. Calculations were carried out using the R system for statistical computing (version 3.4.0; R Development Core Team, Vienna, Austria, 2017).

## Results

### Descriptive analysis

After the exclusion of male patients and patients with distant metastasis at the initial diagnosis, no observation time, local recurrence, or missing values for *CCND1* status, the final study cohort included 511 patients (Fig. 1). Among them, 378 patients (74.0%) had HR-positive, HER2-negative tumors, 74 patients (14.5%) had triple-negative breast cancer (TNBC), and 59 patients (11.5%) had HER2-positive tumors. Patient and tumor characteristics are shown in Table 1.

**Table 1:** Patient and tumor characteristics

Characteristic	All patients (*n* = 511)	HR-positive, HER2-negative patients (*n* = 378)	HR-negative, HER2-negative patients (*n* = 74)	HER2-positive patients (*n* = 59)
Age at diagnosis (y; mean, SD)	57.0 (12.4)	57.9 (12.2)	53.8 (13.4)	55.1 (12.0)
BMI (kg/m^2^; median, IQR)	26.2 (5.0)	26.3 (4.9)	26.2 (5.3)	26.0 (5.7)
Tumor stage	–	–	–	–
T1	257 (51.1)	195 (52.6)	39 (53.4)	23 (39.0)
T2	195 (38.8)	141 (38.0)	29 (39.7)	25 (42.4)
T3	27 (5.4)	22 (5.9)	3 (4.1)	2 (3.4)
T4	24 (4.8)	13 (3.5)	2 (2.7)	9 (15.3)
Lymph-node status	–	–	–	–
N0	292 (57.8)	220 (58.7)	41 (57.7)	31 (52.5)
N +	213 (42.2)	155 (41.3)	30 (42.3)	28 (47.5)
Grading	–	–	–	–
G1	44 (8.7)	43 (11.5)	1 (1.4)	0 (0)
G2	325 (64.2)	270 (72.0)	20 (27.4)	35 (60.3)
G3	137 (27.1)	62 (16.5)	52 (71.2)	23 (39.7)
HER2 status	–	–	–	–
HER2–	452 (88.5)	–	–	–
HER2 +	59 (11.5)	–	–	–
ER status	–	–	–	–
ER–	111 (21.7)	–	–	–
ER +	400 (78.3)	–	–	–
PR status	–	–	–	–
PR–	137 (26.8)	–	–	–
PR +	374 (73.2)	–	–	–

Mean (SD) or median (IQR), where appropriate, are shown for continuous characteristics and frequencies (percentage) for categorical characteristics.

*BMI* body mass index; *ER* estrogen receptor; *HER2* human epidermal growth factor receptor 2; *HR* hormone receptor; *IQR* interquartile range; *PR* progesterone receptor; *SD* standard deviation

In total, 66 patients (12.9%) had *CCND1* amplification and 445 patients (87.1%) did not. The distribution of *CCND1* amplification differed across molecular subtypes (Table 2). The proportion of patients with *CCND1* amplification was highest in those with luminal B–like tumors (34/196, 17.3%) and HER2-positive tumors (13/59, 22.0%) and lowest in triple-negative (2/74, 2.7%) tumors.

**Table 2:** Distribution of molecular subtypes and *CCND1* amplification status, showing frequencies and percentages

Molecular subtype	All patients (*n* = 511)	Patients without *CCND1* amplification (*n* = 445)	Patients with *CCND1* amplification (*n* = 66)
Luminal A–like	182 (35.6)	165 (37.1)	17 (25.8)
Luminal B–like	196 (38.4)	162 (36.4)	34 (51.5)
HER2-positive	59 (11.5)	46 (10.3)	13 (19.7)
TNBC	74 (14.5)	72 (16.2)	2 (3.0)

*HER2* human epidermal growth factor receptor 2; *TNBC* triple-negative breast cancer

### CCND1 amplification and prognosis

**Fig. 4:**
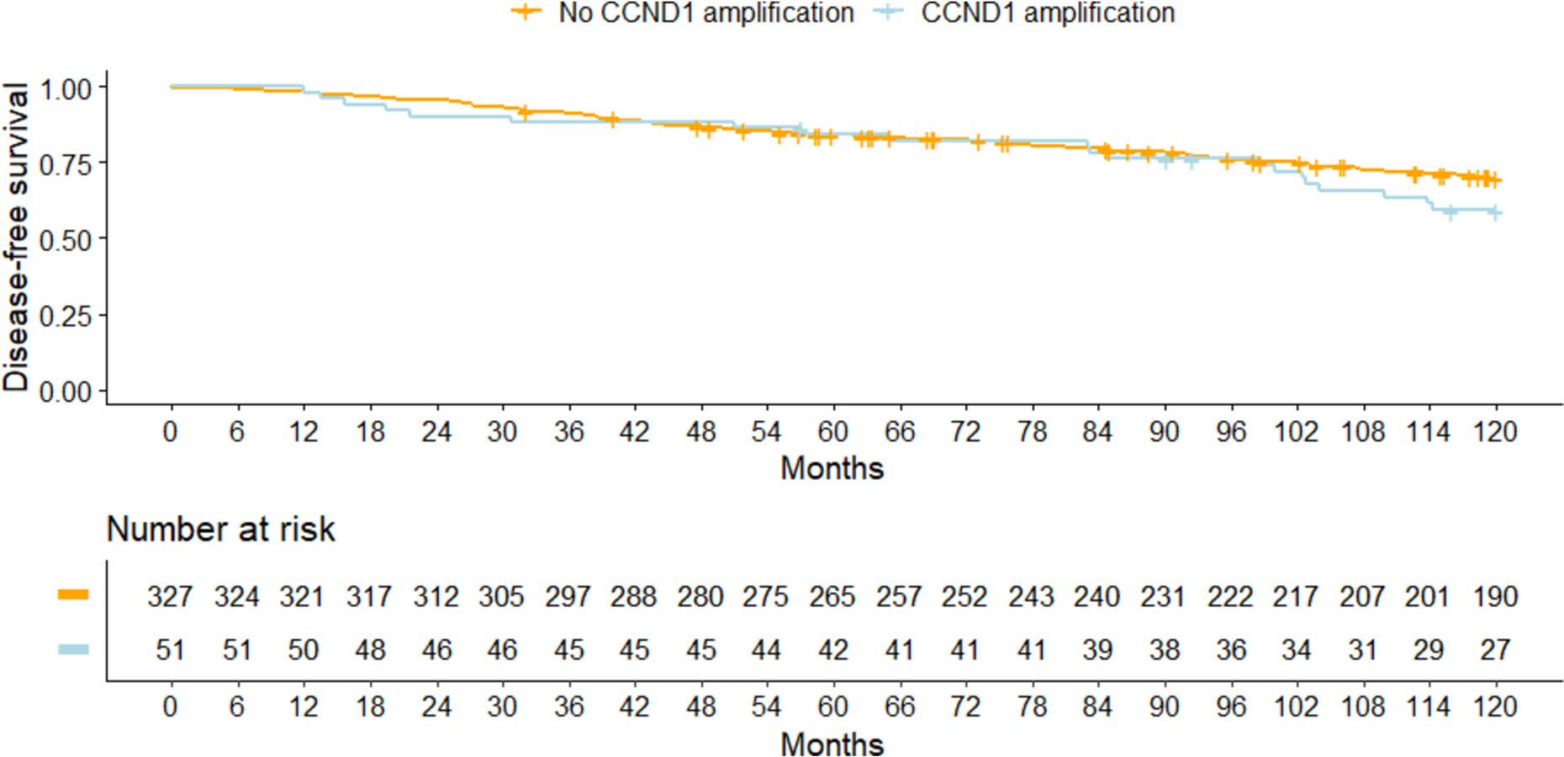
Disease-free survival rates relative to *CCND1* amplification in patients with hormone receptor–positive and HER2-negative tumors

**Fig. 5:**
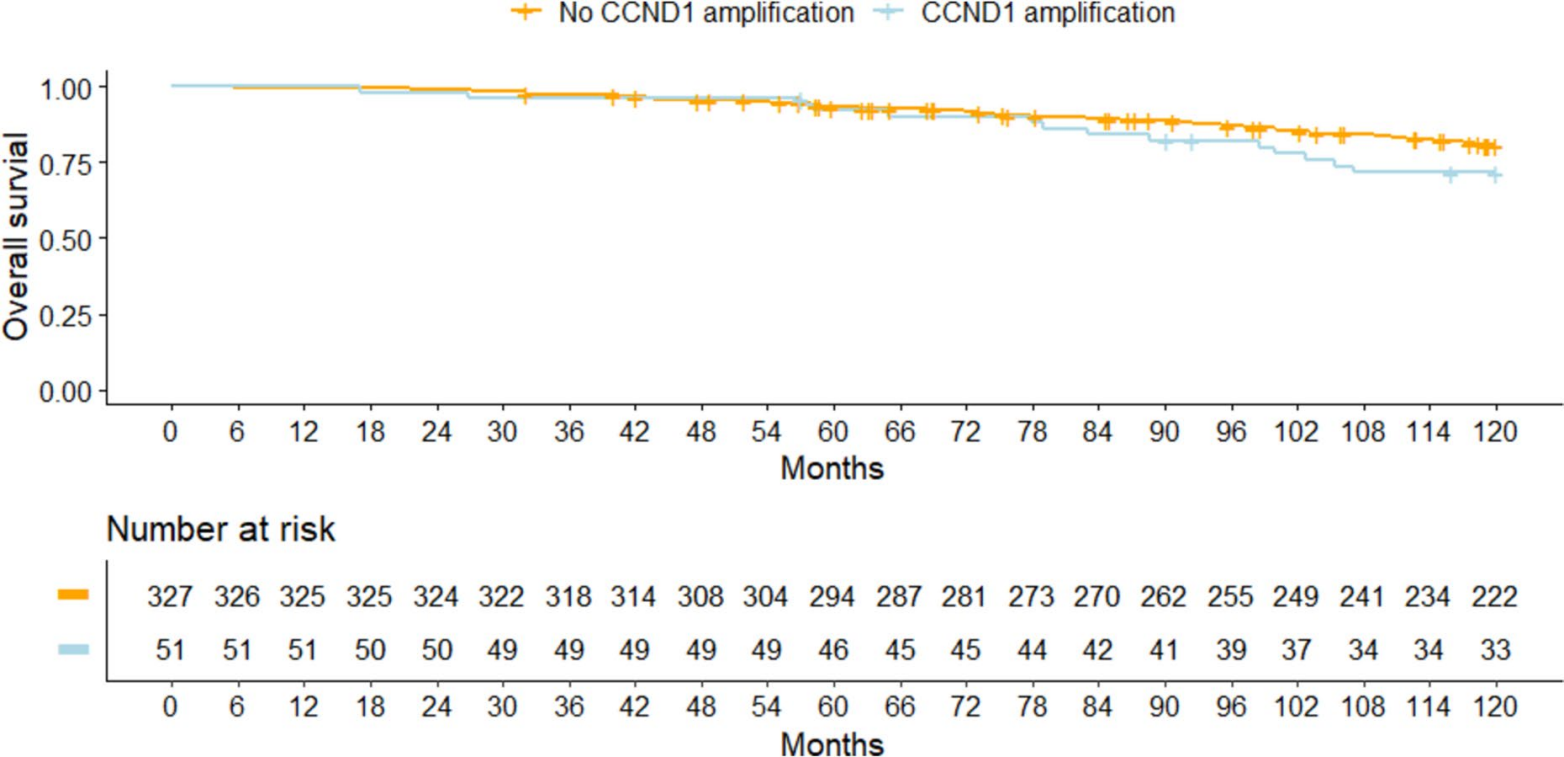
Overall survival rates relative to *CCND1* amplification in patients with hormone receptor–positive and HER2-negative tumors

An influence of *CCND1* amplification on the prognosis in addition to the established prognostic factors could not be identified, either for DFS (*P* = 0.43, LRT) or for OS (*P* = 0.52, LRT). The adjusted hazard ratios for *CCND1* amplification status (yes vs. no) were 1.29 (95% CI, 0.79 to 2.11) for DFS and also 1.30 (95% CI, 0.71 to 2.37) for OS (Table 3). Unadjusted analysis yielded similar results (Tables 3, 4, Figs. 4, 5). The median observation times in patients with HR-positive tumors were 10.0 years (interquartile range [IQR] 6.4–10.0 years) for DFS and 10.0 years (IQR 8.5–10.0 years) for OS. During the observation time, there were 114 DFS events and 73 OS events. The 10-year survival rates for DFS were 0.70 (0.65–0.75) without *CCND1* amplification and 0.59 (0.47–0.75) with amplification. For OS, the 10-year survival rates were 0.81 (0.76–0.85) without *CCND1* amplification and 0.72 (0.60–0.85) with amplification.

**Table 3:** The impact of *CCND1* amplification on the prognosis in patients with hormone receptor–positive, HER2-negative tumors, showing adjusted and unadjusted hazard ratios for disease-free survival (DFS) and overall survival (OS)

Outcome	Adjusted analysis^1^			Unadjusted analysis	
	Hazard ratio (95% CI)	*P* value		Hazard ratio (95% CI)	*P* value
DFS	1.29 (0.79, 2.11)	0.31		1.37 (0.84, 2.22)	0.20
OS	1.30 (0.71, 2.37)	0.40		1.54 (0.86, 2.77)	0.14

CI, confidence intervals; DFS, disease-free survival; OS, overall survival.

^1^Hazard ratios are adjusted for age at diagnosis, body mass index, tumor stage, lymph-node status, and grading

**Table 4:** Survival rates relative to *CCND1* amplification status in patients with hormone receptor–positive, HER2-negative tumors

Outcome	Amplification status	Patients	Events	Survival rate (95% CI)		
				2-year	5-year	10-year
DFS	No *CCND1* amplification	327	94	0.95 (0.93, 0.98)	0.84 (0.80, 0.88)	0.70 (0.65, 0.75)
	*CCND1* amplification	51	20	0.90 (0.82, 0.99)	0.84 (0.75, 0.95)	0.59 (0.47, 0.75)
OS	No *CCND1* amplification	327	59	0.99 (0.98, 1.00)	0.93 (0.90, 0.96)	0.81 (0.76, 0.85)
	*CCND1* amplification	51	14	0.98 (0.94, 1.00)	0.92 (0.85, 1.00)	0.72 (0.60, 0.85)

*CI* confidence intervals; *DFS* disease-free survival; *OS* overall survival

## Discussion

This study investigated *CCND1* amplification in a cohort of patients with early breast cancer, focusing on HR-positive, HER2-negative patients, and the correlation of amplification with outcome and clinicopathologic parameters. The amplification rate in this study was found among unselected breast cancer patients, a figure that is consistent with previously published amplification frequencies ranging between 10 and 15% [29–31]. Although it was a recurring alteration, no statistically significant correlation was observed between *CCND1* amplification status in HR-positive, HER2-negative breast cancer patients and their prognosis. Various studies have reported this lack of correlation [32–35]. Like others, we have shown that *CCND1* amplification status does not add further information about DFS or OS to the established biomarkers of tumor size — lymph-node involvement, and grading [36–38].

Other authors, however, have reported a prognostic value for *CCND1* status. Lundgren et al. noted a higher rate of recurrence in *CCND1*-amplified tumors independent of T, N, grading, and Ki-67 expression (hazard ratio 1.60; 95% CI, 1.08 to 2.41; *P* = 0.03), while Bostner et al. (hazard ratio 1.59; 95% CI, 0.96 to 2.64; *P* = 0.0093), reported an even clearer connection between *CCND1* amplification and a higher risk of recurrence in an ER-positive subset of patients [33, 39]. Lundberg et al. reported a poorer 15-year survival in ER-positive breast cancer patients with *CCND1* amplifications in the Cox proportional hazards model [40]. Other studies have suggested that *CCDN1* amplification may be of greater significance among ER-positive patients [37, 41].

In the present study, *CCND1* amplification was lower among TNBC patients and higher in HER2-positive and HR-positive patients. These figures correspond to the literature findings. A greater frequency of *CCND1* amplifications is observed in ER-positive subsets of breast cancer patients, and a shorter DFS for the ER-positive subset of *CCND1-*amplified patients has been reported [31, 34, 38, 39, 41, 42]. In 2019, Lundberg et al. reported that *CCND1* amplifications—especially in ER-positive, HER2-negative patients without lymph-node involvement—correlated with poor OS [40]. The present study did not find any statistically significant difference in the DFS or OS over a 10-year follow-up period for HR-positive, HER2-negative patients. There may be an impact in different follow-up length, as Lundberg et al. reported a poorer survival after 15 years [40]. But their Kaplan–Meier estimates suggest that survival differences were evident even at much earlier time points. The existing literature indeed presents a heterogeneous landscape regarding the prognostic impact of CCND1 amplification and at what timepoint it occures. For example, Holm et al. reported worse 10-year overall survival in CCND1-amplified luminal A tumors, consistent with the findings of Chin et al., who observed a similar trend with a median follow-up of 6.6 years [16, 43]. Additionally, Bieche et al. demonstrated decreased relapse-free survival within a 10-year period [35]. Conversely, Valla et al. and others found no significant difference in survival at 10 years [32]. Given these mixed findings, further investigation is necessary, particularly in low-risk, HR-positive, node-negative cohorts, to determine whether specific factors can reliably predict late tumor recurrence.

Since *CCND1* appears to play a more important role among HR-positive patients, there has been discussion on whether earlier relapse may be caused by a diminished therapeutic response to tamoxifen. It is still not clear whether cyclin D1 protein overexpression, rather than *CCND1* status, could be used to predict the therapeutic response to tamoxifen, and conflicting results on this have been published [29, 36, 44]. While Quintayo et al. reported that the prognosis is poorer in the presence of *CCND1* amplifications, irrespective of cyclin D1 protein levels, Jirström and coworkers found that the tamoxifen response is influenced by both amplification status and the level of cyclin D1 expression [36].

Endocrine resistance and relapse during treatment with CDK 4/6 inhibitor therapy is the main focus of research for HR-positive patients [45]. Different mechanisms of resistance have been discussed, such as *RB1* mutations and loss of function [46], up-regulation of phosphorylated 3-phosphoinositide-dependent protein kinase 1 (PDK1), which was observed in ribociclib-resistant cell lines [47], or hyperactivity of cyclin A/CDK2, in which cells were found to be able to escape CDK4 loss [48]. Molecular mechanisms would also explain the poorer outcome in *CCND1*-amplified/cyclin D1-overexpressing tumors, leading to increased cyclin D1 expression and inappropriate cyclin D-CDK 4/6 activity, and suggesting that *CCND1* amplification could serve as a predictive biomarker [49, 50]. Data from the PALOMA-1 trial, in cohort two of which only patients with amplifications of cyclin D1 or loss of p16 or both were included, showed no additional benefit for palbociclib in patients with *CCND1*-amplified tumors. Recruitment of this cohort was therefore stopped after an interim analysis [51].

*CCND1* amplification rate in this trial was 12.9% which found by protein expression of cyclin D1 assessed bei FISH analysis. This is according to the literature where in one of the largest examined patient cohorts in the Arimidex, Tamoxifen, Alone or in Combination (ATAC) trial where over 1000 patients where assessed by chromogenic in situ hybridisation (CISH) and immunohistochemistry the ampflification rate was 8.7% [33]. The largest study using SNPs to find *CCND1* amplification reported 22–35% amplification rate [40]. It seems that the method does not correlated to survival as positive and negative results were reported [33, 40, 52, 53]. Nevertheless mRNA levels might provide a more accurate prediction of prognosis in breast cancer patients concerning *CCND1* amplification and this should be further examined.

However, further trials are needed in this field, since patients with luminal B-like tumors in the present study showed an amplification rate of 17.3%. Some data suggest that resistance to CDK 4/6 inhibitors is associated with strong up-regulation of *CCND1* protein levels [54]. A phase 1/phase 2 clinical trial (NCT03901469) examined the combination of a small molecule inhibitor, ZEN-3694, in combination with either palbociclib or abemaciclib to overcome endocrine resistance. The combination was shown to reverse CDK6 and *CCND1* protein levels [54]. No further biomarker analyses have yet been published from CDK 4/6 inhibitor trials; for example, the Natalee trial (NCT03701334) examining ribociclib for adjuvant breast cancer patients will assess *CCND1* as one of the biomarkers. The present study found that in the HER2-positive cohort, 22% of tumors showed *CCND1* amplification, so it may be useful to examine *CCND1* in further trials with triple-positive patients in whom HER2 antibodies are administered in combination with CDK4/6 inhibitors, and to evaluate whether HER2-low tumors also show high *CCND1* amplification rates.

### Limitations

There are no evaluated suggested cutoff values for examining *CCND1* amplification using FISH analysis. *CCND1* amplification was therefore defined as a *CCND1* probe to CEP11 ratio of 2.0 or greater—the same ratio recommended by the U.S. Food and Drug Administration for HER2 amplification diagnosis for breast cancer [26]. Lundgren and coworkers used single nucleotide polymorphism (SNP) arrays to identify amplified versus nonamplified tumors [40]. Jirström et al. also used FISH to evaluate the *CCND1* gene, and it was scored as amplified when the ratio was greater than 1 in at least 20% of tumor cells [44]. Further research is necessary to standardize cutoff values for distinguishing between amplified and nonamplified tumors. Differences in preanalytical and analytical methods with regard to the antibody clones and reading of the immunohistochemistry stains may lead to different results. There are also biological limitations resulting from the use of a tissue microarray, and limitations due to the small sample size of the HR-positive, HER2-negative cohort, in which 378 patients were examined. In addition, *CCND1,* which can be expressed heterogeneously, may be differently interpreted, leading to false-negative results. In addition, the study was conducted on archived tumor tissue and was performed retrospectively.

## Conclusion

FISH analysis of the prognostic value of *CCND1* did not show any significant correlation with DFS and OS in HR-positive, HER2-negative breast cancer patients. Further comprehensive studies are needed in order to understand the impact of *CCND1* amplifications on resulting (downstream) signaling activities and their effects in breast cancer. In particular, the HR-positive cohort with endocrine resistance would benefit from a better understanding of the molecular profile and definition of a more individualized prognostic profile.

## Data Availability

The datasets are available from the corresponding author upon reasonable request.

## References

[1] Ferlay J et al (2013) Cancer incidence and mortality patterns in Europe: estimates for 40 countries in 2012. Eur J Cancer 49(6):1374–1403 23485231 10.1016/j.ejca.2012.12.027

[2] Nadji M et al (2005) Immunohistochemistry of estrogen and progesterone receptors reconsidered: experience with 5,993 breast cancers. Am J Clin Pathol 123(1):21–27 15762276 10.1309/4wv79n2ghj3x1841

[3] Engler T et al (2022) Implementation of CDK4/6 Inhibitors and its influence on the treatment landscape of advanced breast cancer patients—data from the real-world registry PRAEGNANT. Geburtshilfe Frauenheilkd 82(10):1055–1067 36186151 10.1055/a-1880-0087 PMC9525148

[4] Lüftner D et al (2023) Update breast cancer 2022 part 6—advanced-stage breast cancer. Geburtshilfe Frauenheilkd 83(3):299–309 36908287 10.1055/a-2018-9184 PMC9998183

[5] Curtis C et al (2012) The genomic and transcriptomic architecture of 2,000 breast tumours reveals novel subgroups. Nature 486(7403):346–352 22522925 10.1038/nature10983 PMC3440846

[6] Sherr CJ, Roberts JM (1999) CDK inhibitors: positive and negative regulators of G1-phase progression. Genes Dev 13(12):1501–1512 10385618 10.1101/gad.13.12.1501

[7] Ekholm SV, Reed SI (2000) Regulation of G1 cyclin-dependent kinases in the mammalian cell cycle. Curr Opin Cell Biol 12(6):676–684 11063931 10.1016/s0955-0674(00)00151-4

[8] Bartkova J et al (1996) The p16-cyclin D/Cdk4-pRb pathway as a functional unit frequently altered in melanoma pathogenesis. Cancer Res 56(23):5475–5483 8968104

[9] Marino M, Galluzzo P, Ascenzi P (2006) Estrogen signaling multiple pathways to impact gene transcription. Curr Genomics 7(8):497–508 18369406 10.2174/138920206779315737 PMC2269003

[10] Musgrove EA et al (1994) Cyclin D1 induction in breast cancer cells shortens G1 and is sufficient for cells arrested in G1 to complete the cell cycle. Proc Natl Acad Sci U S A 91(17):8022–8026 8058751 10.1073/pnas.91.17.8022 PMC44537

[11] Sherr CJ (1996) Cancer cell cycles. Science 274(5293):1672–1677 8939849 10.1126/science.274.5293.1672

[12] Wang TC et al (1994) Mammary hyperplasia and carcinoma in MMTV-cyclin D1 transgenic mice. Nature 369(6482):669–671 8208295 10.1038/369669a0

[13] Casimiro MC, Arnold A, Pestell RG (2015) Kinase independent oncogenic cyclin D1. Aging (Albany NY) 7(7):455–456 26187783 10.18632/aging.100773 PMC4543029

[14] Abt MC et al (2012) Commensal bacteria calibrate the activation threshold of innate antiviral immunity. Immunity 37(1):158–170 22705104 10.1016/j.immuni.2012.04.011 PMC3679670

[15] Zwijsen RM et al (1997) CDK-independent activation of estrogen receptor by cyclin D1. Cell 88(3):405–415 9039267 10.1016/s0092-8674(00)81879-6

[16] Holm K et al (2012) Characterisation of amplification patterns and target genes at chromosome 11q13 in CCND1-amplified sporadic and familial breast tumours. Breast Cancer Res Treat 133(2):583–594 22002566 10.1007/s10549-011-1817-3

[17] Fasching PA et al (2014) HER2 and TOP2A amplification in a hospital-based cohort of breast cancer patients: associations with patient and tumor characteristics. Breast Cancer Res Treat 145(1):193–203 24682655 10.1007/s10549-014-2922-x

[18] Wöckel, A., et al., *Interdisciplinary Screening, Diagnosis, Therapy and Follow-up of Breast Cancer. Guideline of the DGGG and the DKG (S3-Level, AWMF Registry Number 032/045OL, December 2017) - Part 1 with Recommendations for the Screening, Diagnosis and Therapy of Breast Cancer.* Geburtshilfe und Frauenheilkunde, 2018. **78**(10): p. 927-948. 10.1055/a-0646-4522 PMC6202580 30369626

[19] Wöckel, A., et al., *Interdisciplinary Screening, Diagnosis, Therapy and Follow-up of Breast Cancer. Guideline of the DGGG and the DKG (S3-Level, AWMF Registry Number 032/045OL, December 2017) - Part 2 with Recommendations for the Therapy of Primary, Recurrent and Advanced Breast Cancer.* Geburtshilfe und Frauenheilkunde, 2018. **78**(11): p. 1056–1088. 10.1055/a-0646-4630 PMC6261741 30581198

[20] McShane LM et al (2006) REporting recommendations for tumor MARKer prognostic studies (REMARK). Breast Cancer Res Treat 100(2):229–235 16932852 10.1007/s10549-006-9242-8

[21] Wunderle M et al (2019) Association between breast cancer risk factors and molecular type in postmenopausal patients with hormone receptor-positive early breast cancer. Breast Cancer Res Treat 174(2):453–461 30603996 10.1007/s10549-018-05115-6

[22] Kononen J et al (1998) Tissue microarrays for high-throughput molecular profiling of tumor specimens. Nat Med 4(7):844–847 9662379 10.1038/nm0798-844

[23] Press MF et al (2002) Evaluation of HER-2/neu gene amplification and overexpression: comparison of frequently used assay methods in a molecularly characterized cohort of breast cancer specimens. J Clin Oncol 20(14):3095–3105 12118023 10.1200/JCO.2002.09.094

[24] Sauter G et al (2009) Guidelines for human epidermal growth factor receptor 2 testing: biologic and methodologic considerations. J Clin Oncol 27(8):1323–1333 19204209 10.1200/JCO.2007.14.8197

[25] Wolff AC et al (2013) Recommendations for human epidermal growth factor receptor 2 testing in breast cancer: American society of clinical oncology/college of american pathologists clinical practice guideline update. J Clin Oncol 31(31):3997–4013 24101045 10.1200/JCO.2013.50.9984

[26] Press MF et al (2005) Diagnostic evaluation of HER-2 as a molecular target: an assessment of accuracy and reproducibility of laboratory testing in large, prospective, randomized clinical trials. Clin Cancer Res 11(18):6598–6607 16166438 10.1158/1078-0432.CCR-05-0636

[27] Grambsch PM, Therneau TM (1994) Proportional hazards tests and diagnostics based on weighted residuals. Biometrika 81(3):515–526

[28] Salmen J et al (2014) Pooled analysis of the prognostic relevance of progesterone receptor status in five German cohort studies. Breast Cancer Res Treat 148(1):143–151 25253172 10.1007/s10549-014-3130-4

[29] Li Z et al (2016) Evaluation of CCND1 amplification and CyclinD1 expression: diffuse and strong staining of CyclinD1 could have same predictive roles as CCND1 amplification in ER positive breast cancers. Am J Transl Res 8(1):142–153 27069548 PMC4759424

[30] Karlseder J et al (1994) Patterns of DNA amplification at band q13 of chromosome 11 in human breast cancer. Genes Chromosomes Cancer 9(1):42–48 7507699 10.1002/gcc.2870090108

[31] Elsheikh S et al (2008) CCND1 amplification and cyclin D1 expression in breast cancer and their relation with proteomic subgroups and patient outcome. Breast Cancer Res Treat 109(2):325–335 17653856 10.1007/s10549-007-9659-8

[32] Valla M et al (2022) CCND1 amplification in breast cancer -associations with proliferation, histopathological grade, molecular subtype and prognosis. J Mammary Gland Biol Neoplasia 27(1):67–77 35459982 10.1007/s10911-022-09516-8 PMC9135839

[33] Lundgren K et al (2012) Effects of cyclin D1 gene amplification and protein expression on time to recurrence in postmenopausal breast cancer patients treated with anastrozole or tamoxifen: a TransATAC study. Breast Cancer Res 14(2):R57 22475046 10.1186/bcr3161 PMC3446392

[34] Roy PG et al (2010) High CCND1 amplification identifies a group of poor prognosis women with estrogen receptor positive breast cancer. Int J Cancer 127(2):355–360 19904758 10.1002/ijc.25034

[35] Bieche I et al (2002) Prognostic value of CCND1 gene status in sporadic breast tumours, as determined by real-time quantitative PCR assays. Br J Cancer 86(4):580–586 11870541 10.1038/sj.bjc.6600109 PMC2375286

[36] Quintayo MA et al (2012) GSK3beta and cyclin D1 expression predicts outcome in early breast cancer patients. Breast Cancer Res Treat 136(1):161–168 22976805 10.1007/s10549-012-2229-8

[37] Kirkegaard T et al (2008) Genetic alterations of CCND1 and EMSY in breast cancers. Histopathology 52(6):698–705 18393977 10.1111/j.1365-2559.2008.03007.x

[38] Seshadri R et al (1996) Cyclin DI amplification is not associated with reduced overall survival in primary breast cancer but may predict early relapse in patients with features of good prognosis. Clin Cancer Res 2(7):1177–1184 9816285

[39] Bostner J et al (2007) Amplification of CCND1 and PAK1 as predictors of recurrence and tamoxifen resistance in postmenopausal breast cancer. Oncogene 26(49):6997–7005 17486065 10.1038/sj.onc.1210506

[40] Lundberg A et al (2019) The long-term prognostic and predictive capacity of cyclin D1 gene amplification in 2305 breast tumours. Breast Cancer Res 21(1):34 30819233 10.1186/s13058-019-1121-4 PMC6394106

[41] Al-Kuraya K et al (2004) Prognostic relevance of gene amplifications and coamplifications in breast cancer. Cancer Res 64(23):8534–8540 15574759 10.1158/0008-5472.CAN-04-1945

[42] Hui R et al (1996) Cyclin D1 and estrogen receptor messenger RNA levels are positively correlated in primary breast cancer. Clin Cancer Res 2(6):923–928 9816251

[43] Chin K et al (2006) Genomic and transcriptional aberrations linked to breast cancer pathophysiologies. Cancer Cell 10(6):529–541 17157792 10.1016/j.ccr.2006.10.009

[44] Jirstrom K et al (2005) Adverse effect of adjuvant tamoxifen in premenopausal breast cancer with cyclin D1 gene amplification. Cancer Res 65(17):8009–8016 16140974 10.1158/0008-5472.CAN-05-0746

[45] Nabieva N, Fasching PA (2023) CDK4/6 inhibitors—overcoming endocrine resistance is the standard in patients with hormone receptor-positive breast cancer. Cancers 15(6):1763 36980649 10.3390/cancers15061763 PMC10046117

[46] Guarducci C et al (2017) Mechanisms of resistance to CDK4/6 inhibitors in breast cancer and potential biomarkers of response. Breast Care (Basel) 12(5):304–308 29234249 10.1159/000484167 PMC5704709

[47] Jansen VM et al (2017) Kinome-wide RNA interference screen reveals a role for PDK1 in acquired resistance to CDK4/6 inhibition in ER-positive breast cancer. Cancer Res 77(9):2488–2499 28249908 10.1158/0008-5472.CAN-16-2653 PMC5421398

[48] Patel P et al (2018) Dual inhibition of CDK4 and CDK2 via targeting p27 tyrosine phosphorylation induces a potent and durable response in breast cancer cells. Mol Cancer Res 16(3):361–377 29330290 10.1158/1541-7786.MCR-17-0602 PMC5835198

[49] Musgrove EA et al (2011) Cyclin D as a therapeutic target in cancer. Nat Rev Cancer 11(8):558–572 21734724 10.1038/nrc3090

[50] Jeffreys SA, Becker TM, Khan S, Soon P, Neubauer H, de Souza P, Powter B (2022) Prognostic and predictive value of CCND1/Cyclin D1 amplification in breast cancer with a focus on postmenopausal patients: a systematic review and meta-analysis. Front Endocrinol 13:895729 10.3389/fendo.2022.895729 PMC9249016 35784572

[51] Finn RS et al (2015) The cyclin-dependent kinase 4/6 inhibitor palbociclib in combination with letrozole versus letrozole alone as first-line treatment of oestrogen receptor-positive, HER2-negative, advanced breast cancer (PALOMA-1/TRIO-18): a randomised phase 2 study. Lancet Oncol 16(1):25–35 25524798 10.1016/S1470-2045(14)71159-3

[52] Hwang TS et al (2003) Prognostic value of combined analysis of cyclin D1 and estrogen receptor status in breast cancer patients. Pathol Int 53(2):74–80 12588434 10.1046/j.1440-1827.2003.01441.x

[53] Gillett C et al (1996) Cyclin D1 and prognosis in human breast cancer. Int J Cancer 69(2):92–99 8608989 10.1002/(SICI)1097-0215(19960422)69:2<92::AID-IJC4>3.0.CO;2-Q

[54] Kharenko OA et al (2022) Combination of ZEN-3694 with CDK4/6 inhibitors reverses acquired resistance to CDK4/6 inhibitors in ER-positive breast cancer. Cancer Gene Ther 29(6):859–869 34385584 10.1038/s41417-021-00375-9

